# Piezo1 facilitates the initiation and progression of renal fibrosis by mediating cell apoptosis and mitochondrial dysfunction

**DOI:** 10.1080/0886022X.2024.2415519

**Published:** 2024-11-04

**Authors:** Yanping Zhang, Lei Lv, Zhaokai Zhou, He Zhang, Qi Li, Shuai Yang, Yibo Wen, Qingwei Wang, Jinjin Feng, Wei Lu, Wei Jia, Jian Guo Wen

**Affiliations:** a Urodynamic Centre, Henan Joint International Pediatric Urodynamic Laboratory and Department of Urology, The First Affiliated Hospital of Zhengzhou University, Henan, China; b Department of Urology, Xinyang Central Hospital, Xinyang, Henan, China; c Department of Urology, Guangzhou Women and Children’s Medical Center, Guangzhou Medical University, Guangdong, China

**Keywords:** Chronic kidney disease, renal fibrosis, Piezo1, apoptosis, mitochondrial dysfunction

## Abstract

Renal fibrosis is the major pathological changes of Chronic kidney disease (CKD). Piezo1, a mechanical sensitive ion channel, is implicated in organ fibrosis. However, the precise role of Piezo1 in CKD fibrosis is unknown. The aims of this study were to identify that the role of Piezo1 in CKD fibrosis and its potential involvement of mitochondrial dysfunction. We performed the study with the Piezo1 agonist Yoda1, Bax inhibitor BAI1, Piezo1 inhibitor GsMTx4 and detected the injury, fibrosis, apoptosis markers and mitochondrial dysfunction. The results showed that the levels of apoptosis, mitochondrial dysfunction, injury and fibrosis increased in TCMK-1 cells after treatment with Yoda1. However, these changes that induced by Yoda1 were relieved by BAI1. Similarly, inhibition Piezo1 with GsMTx4 also partly relieved the renal injury, renal fibrosis, apoptosis and mitochondrial dysfunction *in vivo* and vitro. In conclusion, we found Piezo1 promoted the initiation and development of renal fibrosis and inhibiting Piezo1 improved the fibrosis.

## GRAPHICAL ABSTRACT

**Figure:**
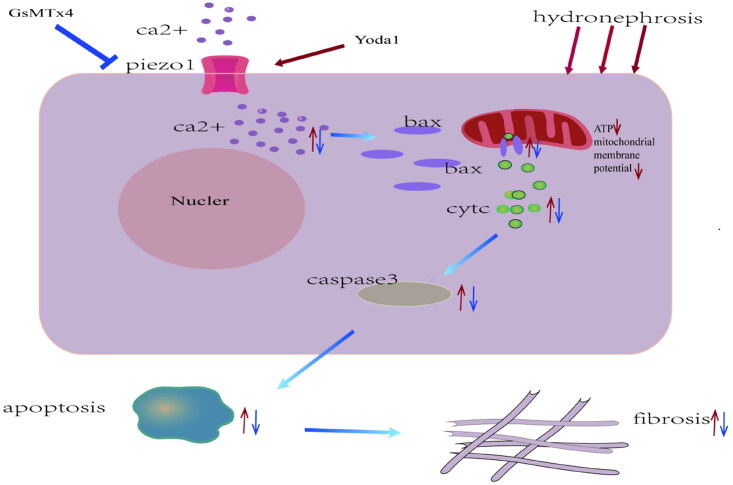

**The signal path diagram of Piezo1 inducing real injury and fibrosis.** The expression of mechanically sensitive channel Piezo1 increased in renal proximal tubular epithelial cells during hydronephrosis. The increased Piezo1 permeated extracellular Ca2+ into intracellular, then facilitated the translocation of Bax to the mitochondrial membrane, which decreased mitochondrial membrane potential and leaded to Cytc leakage, and facilitated the apoptosis, then induced the fibrosis. Inhibition Piezo1 using GsMTx4 can improve the apoptosis and fibrosis.

## Introduction

Chronic kidney disease (CKD) is becoming a public health issue worldwide [1]. It may become the fifth cause of death worldwide by 2040 [2]. Different etiologies (such as genetics, sex, autoimmune related infections, environmental factors, diet, drugs and obstructions) can lead to different degrees of CKD and initial CKD lesions, but the common pathological change is renal tubule interstitial fibrosis (TIF) [3]. TIF is not only a sign and common pathway of various types of progressive CKD but also a major determinant and reliable prognostic indicator of renal failure [4]. Therefore, the keys to treating CKD are the effective prevention or reversal renal fibrosis.

Apoptosis is a programmed death process associated with a variety of primary and secondary kidney diseases. A previous study highlighted the significant role of renal tubular cell apoptosis in the progression of CKD [5]. Mitochondria are closely related to apoptosis. When stimulated, mitochondria dysfunction can occur resulting in an increase in ROS, a decrease in ATP, a decrease in the mitochondrial membrane potential and leakage of mitochondrial contents, leading to disease occurrence [6]. Bax, a proapoptotic factor, can translocate to mitochondria, which leads to an increase in mitochondrial outer membrane permeability (MOMP), the leakage of mitochondrial contents, and ultimately cell apoptosis [7,8]. Mitochondrial dysfunction can accelerate the renal fibrosis [9,10], and targeting mitochondria protects renal function and decreases renal fibrosis [11]. Thus, mitochondrial intervention is an effective measure to reverse the progression of renal fibrosis.

Urinary tract obstruction is a common cause of CKD, and unilateral ureteral obstruction (UUO) is a common research model of renal fibrosis. During UUO, urine flow is blocked and regurgitates into renal tubules, in which renal tubule epithelial cells (TECs) are subjected to mechanical stimulation. After mechanical stimulation, TECs injury occurs, followed by apoptosis, leading to TIF [12]. Therefore, mechanical stimulation of TECs may be an effective target for inhibiting or alleviating the initiation and development of renal fibrosis. Mechanical sensitive cation channels(MSCs) can translate mechanical cues into biological signals and activate intracellular signaling pathways [13]. Piezo1, a newly discovered mechanosensitive channel (MSC), has been reported to play essential roles in various tissues, especially those that are highly exposed to mechanical stimuli, such as the colon, kidneys, skin, bladder and lungs [14]. Piezo1 converts mechanical signal into electrostimulation signals by permeating extracellular Na+, K+, Ca2+ and Mg2+, and preferentially Ca2+ into intracellular, which leads to a series of cell behaviors, such as spreading, migration, proliferation, differentiation, damage and apoptosis [15,16]. Recently, several studies revealed the association between Piezo1 and apoptosis. Studies have shown that Piezo1 overexpression contributes to the apoptosis of type II pneumocytes [17], and that ShRNA‐Piezo1 protects cells by reducing mitochondrial dysfunction and nucleus pulposus cell apoptosis [18]. Similarly, many studies have investigated the relationships between Piezo1 and different types of fibrosis, such as pulmonary fibrosis [19], skin fibroproliferative disease [20], cardiac fibrosis [21], pancreatic fibrosis [22], and renal fibrosis [23–25]. Although Piezo1 is crucial in renal fibrosis, whether Piezo1 can induce or accelerate the renal fibrosis through facilitating mitochondrial dysfunction and the renal tubular epithelial cell apoptosis is unknown.

Consequently, the aim of this study was to investigate the relationships among Piezo1, mitochondrial dysfunction, and apoptosis in the initiation and progression of kidney fibrosis and to explored a new mechanism for renal fibrosis.

## Materials and methods

### Experimental design

Male C57BL/6 mice, aged 10–12 weeks and weighing 25 ± 2 g, were obtained from the animal center of Zhengzhou University and housed in cages, at a suitable temperature (23 ± 3 °C) and a 12h on/off light cycle, and had free access to water and food. The mice were divided randomly into four groups: sham, UUO 3d, UUO 7d and UUO 14d groups. The sham group had only separated ureters, whereas the other groups had bound left ureters. Renal and blood samples were collected at a specific times for subsequent experiments.

To determine whether Piezo1 plays an important role in UUO-induced kidney fibrosis, the mice were divided into 4 groups: control group, 7d UUO group, GsMTx4 group, and 7d UUO+GsMTx4 group. In the GsMTx4 and UUO+GsMTx4 groups, the mice were intraperitoneally injected with 1 mg/kg GsMTx4 every other day starting on the day of the UUO surgery. Mice in the control group received the same volumes of saline. The mice were sacrificed after 7 days, and the kidneys were obtained for subsequent analysis.

Five patients with obstruction kidneys and 5 patients with renal carcinoma who were treated at the First Affiliated Hospital of Zhengzhou University during the period from October of 2023 to May of 2024 were enrolled in this study. Samples from patients with obstructed kidneys were obtained from nonfunctional renal with hydrops, and the control samples were obtained from renal tissue away from the renal carcinoma. The basic characteristics of the patients are listed in the supplementary materials (Supplementary Table 1).

### Measurement of BUN and SCR concentrations

Blood samples were collected and centrifuged at 3000 rpm for 15 min. Blood urea nitrogen (BUN) and serum creatinine (SCr) concentrations were measured using a urea assay kit(S03036) and a creatinine assay kit(S03076), respectively. BUN and SCr concentrations were measured with a Chemray 800 automatic biochemical analyzer (Shenzhen Leidu Life Technology, China). SCr and BUN concentrations in patients were obtained from clinical case data after obtaining patient consent.

### HE and Masson staining

Kidney tissue was fixed with 4% neutral formaldehyde, then embedded in paraffin and sliced into 4 μm tissue slices. The tissue slices were stained following standard HE or Masson staining procedures. The morphological characteristics of the renal tissue and the Masson staining results were observed under a microscope. Tubule injury was evaluated according to the degree of tubular dilatation, absence of brush border, tubular necrosis, and tubular pattern formation, and tubular injury scores were rated on a scale of 0–4 (0, normal; 1, <25%; 2, 25–50%; 3, 50–75%; and 4, ≥75%) [26].

### Western blotting

RIPA buffer (Servicebio, G2002) was used to extract protein samples. The total protein concentration was measured using the BCA assay (Seven, sw101). Proteins were separated *via* 7%,10% or 12% SDS-PAGE and transferred to a 0.45 or o.22um PVDF membrane. The membranes were subsequently blocked with 5% BSA at room temperature for 2h. The membranes were incubated with primary antibodies overnight at 4 °C. The next day, the membranes were incubated with the secondary antibodies. The proteins were visualized *via* enhanced chemiluminescence (ECL) and analyzed with Image J software. The following antibodies were used Piezo1(Proteintech,15939-1-AP, 1:1000), Bcl-2 (Proteintech, 68103-1-Ig, 1:1000), Bax (Proteintech, 50599-2-Ig,1:1000), alpha-smooth muscle actin (α-SMA) (Abcam, ab32575, 1:1000), Fn(Servicebio,GB114491,1:1000), CollagenI (Servicebio, GB11022, 1:1000), Caspase3 (CST, 9662S, 1:1000), Cleaved-caspase3 (CST, 9661S, 1:1000), Cytc (Proteintech, 12245-1-AP, 1:1000), KIM1 (Boster, ba3537, 1:1000), NGAL (Boster, ba9609, 1:1000), Tubulin (ZENBIO, 380628, 1:5000), GAPDH(ZENBIO, 380626, 1:5000), HRP-Anti-Rabbit lgG (ZENBIO, 511203, 1:5000), HRP-Anti-mouse lgG (ZENBIO, 511103, 1:5000).

### Cell culture and intervention

The TCMK-1 cell line was obtained from Servicebio. The cells were cultured in DMEM/high glucose supplemented with 10% FBS and 1% penicillin-streptomycin in a humidified incubator with 5% CO2 at 37 °C. Intervention experiments were performed when the cells had been cultured to a confluence of 70%-80%. Each group was treated with Yoda1 (MedChemExpress, HY-18723, China), BAI1 (MedChemExpress, HY-103269, China), or GsMTx4 (MedChemExpress, HY-P1410, China)(4um) for 24h before mRNA and protein were extracted.

### Real-time PCR

TRIzol (Servicebio,G3013,Wuhan) was used to extract total RNA from tissue and cells following the manufacturer’s instructions. Complementary DNAs (cDNAs) was synthesized using the PrimeScript^™^ RT Master Mix (Takara,RR047A, Japan), after which the RNA concentration was measured using Nanodrop 3000. Quantitative PCR was performed using TB Green^®^ Premix Ex Taq^™^ II (Tli RNaseH Plus) (Takara, RR820A, Japan) and a Bio-Rad CFX384 Real-Time System. The mRNA levels were normalized to those of β-actin. The relative expression was calculated *via* the 2^–ΔΔCt^ method. The relevant primer sequences are shown in Supplementry Table 2.

### Cell viability assay

Cell viability assays was tested using cell counting kit-8 (CCK-8) (MedChemExpress, HY-K0301, China) according to the manufacturer’s instructions. Cells were seeded in 96-well plates and treated with different concentrations of Yoda1, BAI1 and GsMTx4. The CCK-8 solutions were added to cells, follows by incubation for 3h. Absorption at 450 nm was subsequently measured on the molecular devices (SpectraMax i3x, United States). Each experiment was repeated three times. The results are shown in Supplementary Fig1.

### Immunofluorescence

For mouse and human tissue immunofluorescence staining, 4 µm sections were dewaxed with xylene and placed in sodium citrate buffer (pH 6.0) for antigen repair and peroxidase blocking with H_2_O_2_. The cells were fixed for 10 min in 4% paraformaldehyde, permeabilised with 0.5% Triton X-100 and blocked with 5% goat serum. The sections were incubated with primary antibodies, against Piezo1(Proteintech,15939-1-AP, 1:200), AQP1(Abcam, ab15080, 1:3000), α-SMA(Abcam, ab32575, 1:200), and Collagen I (Servicebio, GB11022, 1:200) overnight at 4 °C. Then the sections were incubated with the FITC-TSA (Servicebio, GB1223, 1:500) for 10 min or CY3-labeled goat anti-rabbit IgG (Servicebio, GB21303, 1:300) or anti-rabbit IgG (Alexa Fluor 488) (Servicebio, GB25303, 1:400) for 1h. Next, the sections were stained with DAPI (Beyotime, C1002, China), and the images were obtained using a fluorescence microscope (Leica, C1002, Germany).

### TUNEL staining

A fluorescein isothiocyanate (FITC) TUNEL Cell Apoptosis Detection Kit (Servicebio, G1501-50T, China) was used to detect apoptotic in kidney tissue. Kidney tissue was treated with 4% PEA for 12 h. The tissue was dehydrated, embedded in paraffin, and sliced. In accordance with the TUNEL cell apoptosis detection kit, the tissue was deparaffinized and permeabilized, and a mixture consisting of TDT enzyme, dUTP and buffer at a 1:5:50 ratio was added to the sections, followed by incubation at 37 °C for 1h.The nucleus was stained with DAPI. Then, TUNEL-positive cells were detected *via* by fluorescence microscopy.

### Transmission electron microscopy

A transmission electron microscope was used to observe the mitochondrial ultrastructure. Kidney samples were prefixed in cacodylate buffer (0.1 M, pH 7.4), in which paraformaldehyde (2.5%) and glutaraldehyde (2.5%) were included. The samples were subsequently transferred into osmium tetraoxide (1%) and incubated at 4 °C for 1h. After gradient dehydration in alcohol, the samples were oriented longitudinally, embedded in Epon 812, and cut into 70 nm thick sections, which were contrasted with lead citrate and uranyl acetate. A transmission electron microscope at 80 kV (JEO Ltd.) was used to analyze these prepared sections.

### Apoptosis assay

TCMK-1 cells were digested with trypsin without EDTA according to the instructions of the Annexin V-PE/7-ADD Apoptosis Detection Kit (KeyGEN BioTECH, Cat#KGA1015, China). Then, the cells were washed twice with PBS, and approximately 1 × 10^5^ cells were collected and stained with a 500 µL binding buffer containing 1 µL of annexin V-PE and 5 µL of 7-AAD. Apoptosis was assayed *via* flow cytometry machine (BD Biosciences, Shanghai, China).

### Mitochondrial membrane potential determination (MMP)measurement

JC-1 (Beyotime,C2006, China) was used to measure the MMP, and the experiment was conducted according to the manufacturer’s instructions. JC-1 aggregates and monomers were detected using a flow cytometry machine (BD Biosciences, Shanghai, China) or fluorescence microscope.

### Calcium imaging

TCMK-1 cells were seeded on 12-well plates for 24 h, after which the cells were treated with Yoda1(4 µm), TGFβ1(10 ng/ml) (MedChemExpress, HY-P7117, China) and/or GsMTx4 (4 µm) for 24 h. The intracellular calcium concentration was measured with a Fluo-4 calcium assay kit (Beyotime, S1061S, China) and fluorescence microscopy.

### Measurement of ATP concentration

The ATP concentration was determined using an Enhanced ATP Assay Kit (Beyotime, S0027, China) according to the manufacturer’s instructions. The protein concentration was determined with a BCA protein concentration determination kit (Beyotime, P0009, China), and the concentration of ATP was converted to the nmol/mg protein.

### Statistical analysis

The results of the experiments were assessed from three biological and technical repeats. All the data are presented as the means ± standard deviations (mean ± SDs). Multiple comparisons were evaluated *via* one-way ANOVA followed by Bonferroni’s *post hoc* test. Student’s *t*-test was used to compare the two datasets. *p* < 0.05 was considered to indicate statistical significance.

## Results

### Renal injury and renal fibrosis in the UUO model

**Figure 1.**
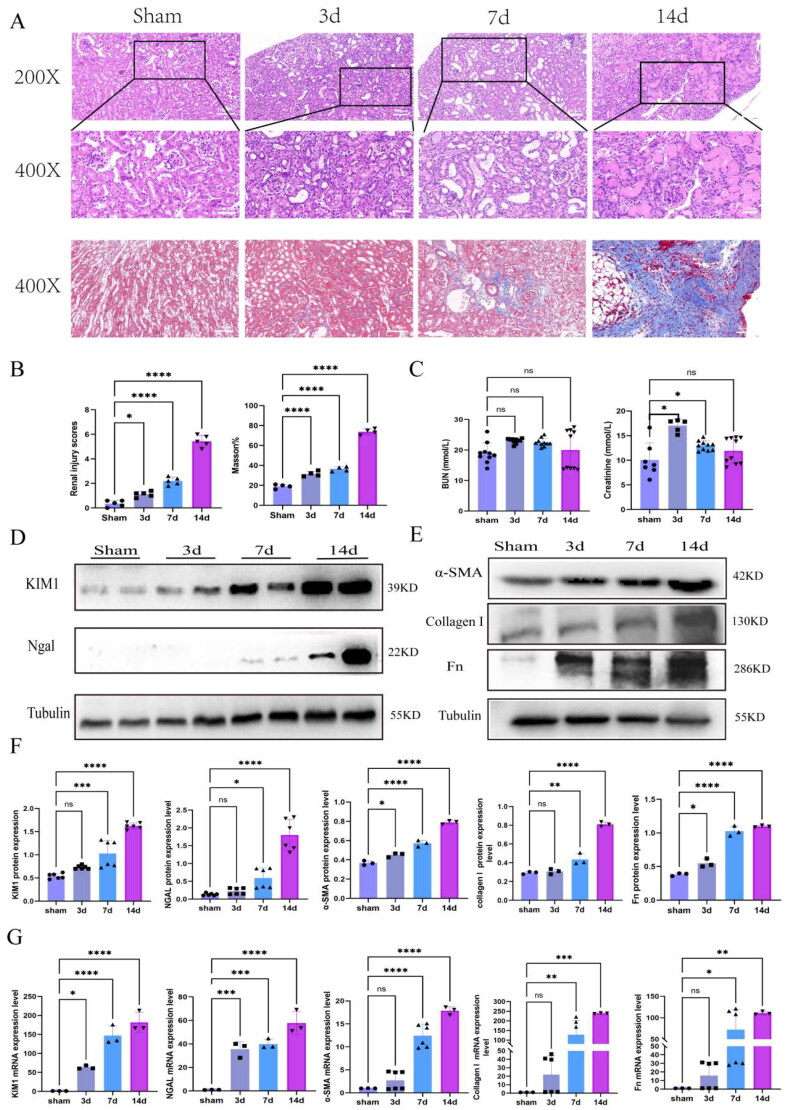
Renal injury and renal fibrosis in UUO mice model. (A) The staining of H&E and Masson in UUO model. Scale bar =100µm. (B) The quantitative analysis of tubule injury and collagen deposition in UUO model (*n* = 5 mice per group). (C) The change of BUN and Creatinine in UUO model. (D) Western blot analysis of NGAL and KIM1 in UUO mice model (*n* = 5 mice per group). (E) Western blot analysis of α-SMA, Collagen I and Fn in UUO mice model (*n* = 5 mice per group). (F) The quantitative analysis result of NGAL, KIM1, α-SMA, Collagen I and Fn proteins (*n* = 5 mice per group). (G) The mRNA quantitative analysis results of NGAL, KIM1, α-SMA, Collagen I and Fn. Statistical analyses were analyzed by one-way ANOVA. *p ≤ 0.05, **p ≤ 0.01, ***p ≤ 0.001, ****p ≤ 0.0001.

We established a UUO mouse model, and renal histopathology and molecular analysis revealed kidney fibrosis. HE staining revealed that kidneys of UUO model mice exhibited tubular dilation, debris in the tubular lumen space, tubular structural failure and inflammatory cell infiltration (Figure 1A). Renal tubular injury scores were analyzed, and the degree of injury in the UUO group was greater than in the sham group (Figure 1B). Masson staining showed that the collagen deposition was aggravated (Figure 1A), the results of the quantitative analysis of Masson were showed in Figure 1B. Besides, we also examined the blood urea nitrogen (BUN) and creatinine levels in mouse serum, and found that the BUN levels was not altered compared with that in the sham group. However, the creatinine levels increased in the UUO 3d and 7d groups, but the 14d group returned to baseline level (Figure 1C). These findings demonstrated that the healthy kidneys had significant compensatory power. Furthermore, we assessed the markers of injury and fibrosis. The WB and PCR showed that the KIM1 and NGAL expression levels were increased in the UUO group than in the sham group (Figure 1D and G). Similarly, the protein and mRNA expression levels of α-SMA, Collagen I and Fn in the UUO group were greater than those in the sham group (Figure 1E and G). The results of the quantitative analysis of renal injury and fibrosis-related proteins were shown in Figure 1F.

### Renal injury and renal fibrosis in obstructed kidney specimens

**Figure 2.**
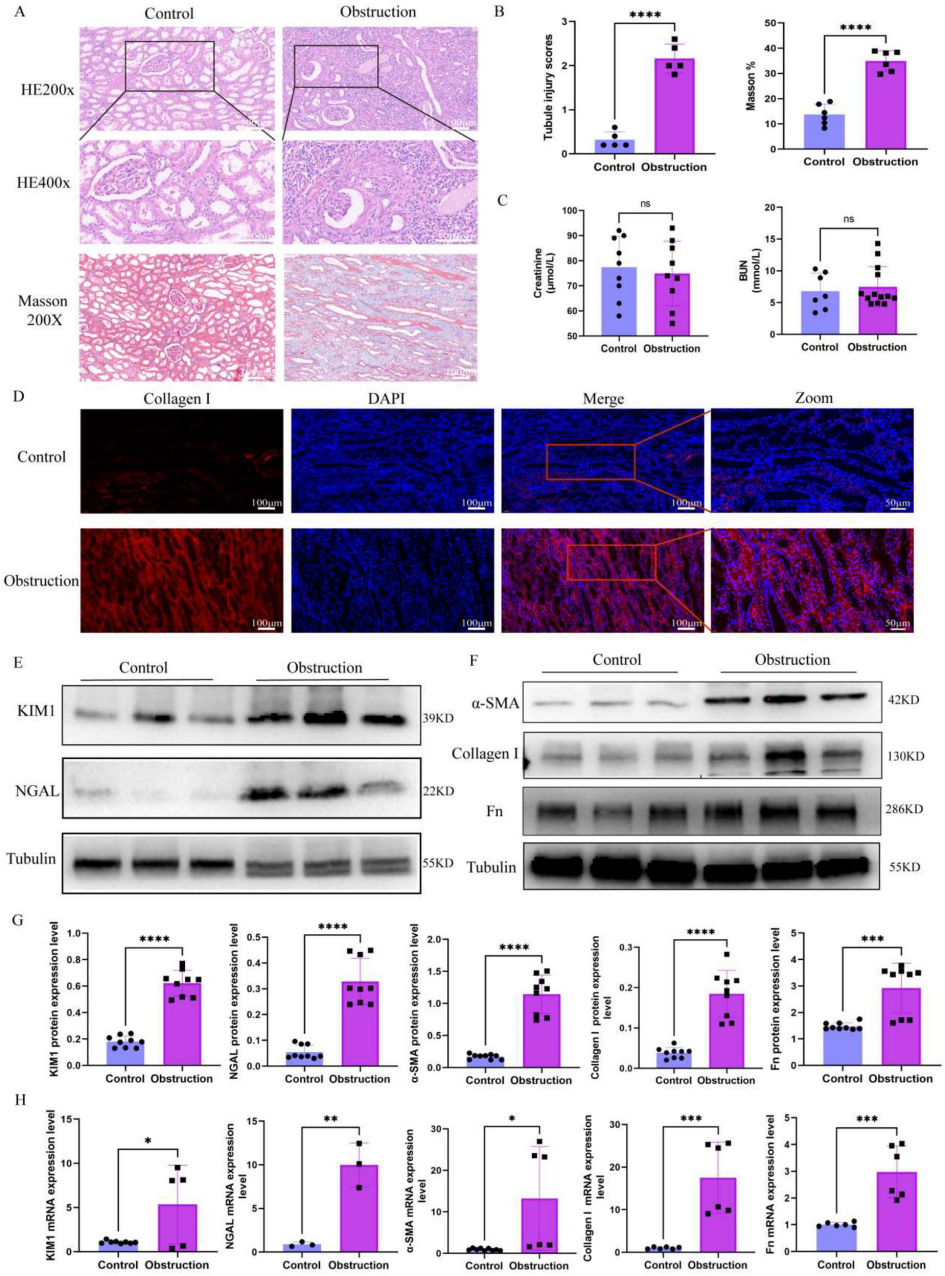
Renal injury and renal fibrosis in human obstructed kidney specimens. (A) H&E and Masson of obstructed kidney specimen sections. Scale bar =100µm. (B) Quantification of the mean intensity of tubule injury and Masson staining. (*n* = 6 specimens per group, 6 random sights per specimens). (C) The level of creatinine and BUN in obstructed kidney patients (*n* = 10 per group). (D) Immunofluorescence staining of collagen I in human obstructed kidney specimen sections (6 random sights per specimens). (E) Western blot analysis of Kim1and NGAL in human obstructed kidney specimens (*n* = 6 specimens per group). (F) Western blot analysis of α-SMA, collagen I and Fn in human obstructed kidney specimens (*n* = 6 specimens per group). (G) Bar plot represents the quantification of relative protein expression levels. (H) Relative mRNA levels of KIM1, NGAL, α-SMA, collagen I and Fn in human obstructed kidney specimens. Statistical analyses were calculated using 2-tailed student’s t test for 2 groups. *p ≤ 0.05, **p ≤ 0.01, ***p ≤ 0.001, ****p ≤ 0.0001.

We obtained kidney specimens from patients and performed relevant tests. HE and Masson staining showed tubular dilation, tubular structural failure, inflammatory cell infiltration and collagen deposition (Figure 2A and B). The creatinine and BUN levels were not different between the control and obstruction groups (Figure 2C). Compared with that in the control group, collagen I immunofluorescence also revealed collagen deposition in the obstruction group (Figure 2D). Similarly, we also measured the injury and fibrosis markers. The results showed the protein levels of KIM1, NGAL, α-SMA, Collagen I and Fn were higher in the obstruction group than control group (Figure 2E and F). The protein and mRNA quantitative analysis results were showed in Figure 2G and H.

### The expression of Piezo1 increased in UUO model mice and obstructed kidney specimens of humans

**Figure 3.**
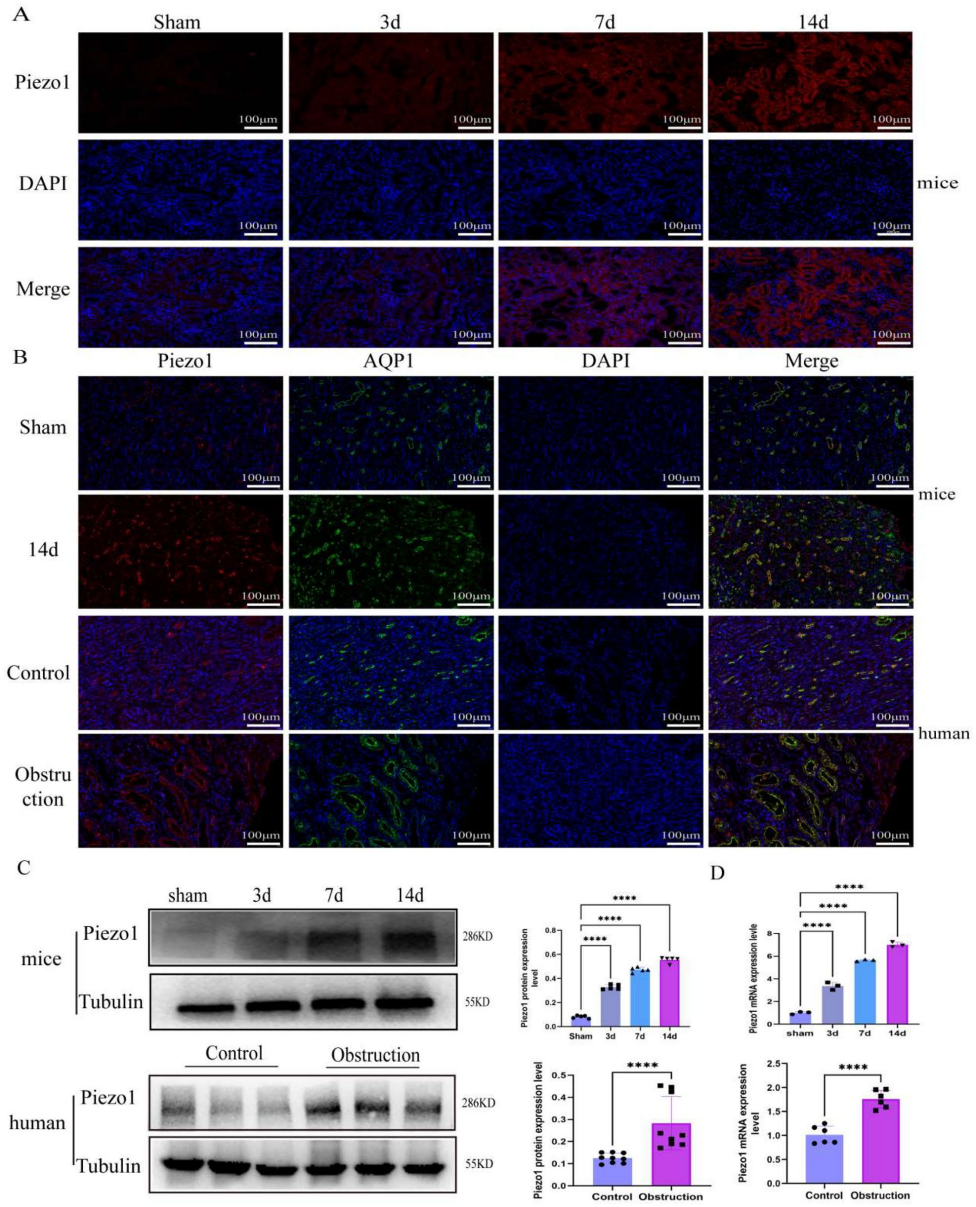
The expression of Piezo1 in mice UUO model and human obstructed kidney specimens. (A) Immunofluorescence staining of Piezo1 in mice UUO model, red represent the expression of Piezo1, blue is the nucleus (*n* = 5 mice per group, 4 random sights per mice). (B) the immunofluorescence staining of Piezo1 (red) and AQP1 (green) colocalization in the proximal tubules of UUO mice and human obstruction kidney (*n* = 5 per group, 6 random sights per group). (C) the WB result and protein quantification of Piezo1 in mice UUO model and human obstructed kidney specimens (*n* = 5 per group, 6 random sights per group). (D) mRNA quantification of Piezo1 in mice UUO model and human obstructed kidney specimens. The data are represented as mean ± SD, statistical analyses were analyzed by one-way ANOVA or 2-tailed student’s t test. *p ≤ 0.05, **p ≤ 0.01, ***p ≤ 0.001, ****p ≤ 0.0001. Scale bar 100 μm.

We assessed the expression of Piezo1 in the kidney to investigate whether Piezo1 was essential in renal fibrosis. Immunofluorescence staining of Piezo1 in the UUO model mice showed that Piezo1 expression increased with the injury and fibrosis development (Figure 3A). AQP1, a marker of renal proximal tubules, co-localizing with Piezo1, as determined by immunofluorescence, indicated that Piezo1 was expressed in mouse and human kidney proximal tubules (Figure 3B). Similarly, we also assessed the expression of Piezo1 protein and gene level in obstructed specimens from humans and in UUO model mice, and found that Piezo1 expressions were higher (Figure 3C and D).

### Apoptosis and mitochondrial dysfunction in UUO model mice and obstructed kidney specimens from humans

**Figure 4.**
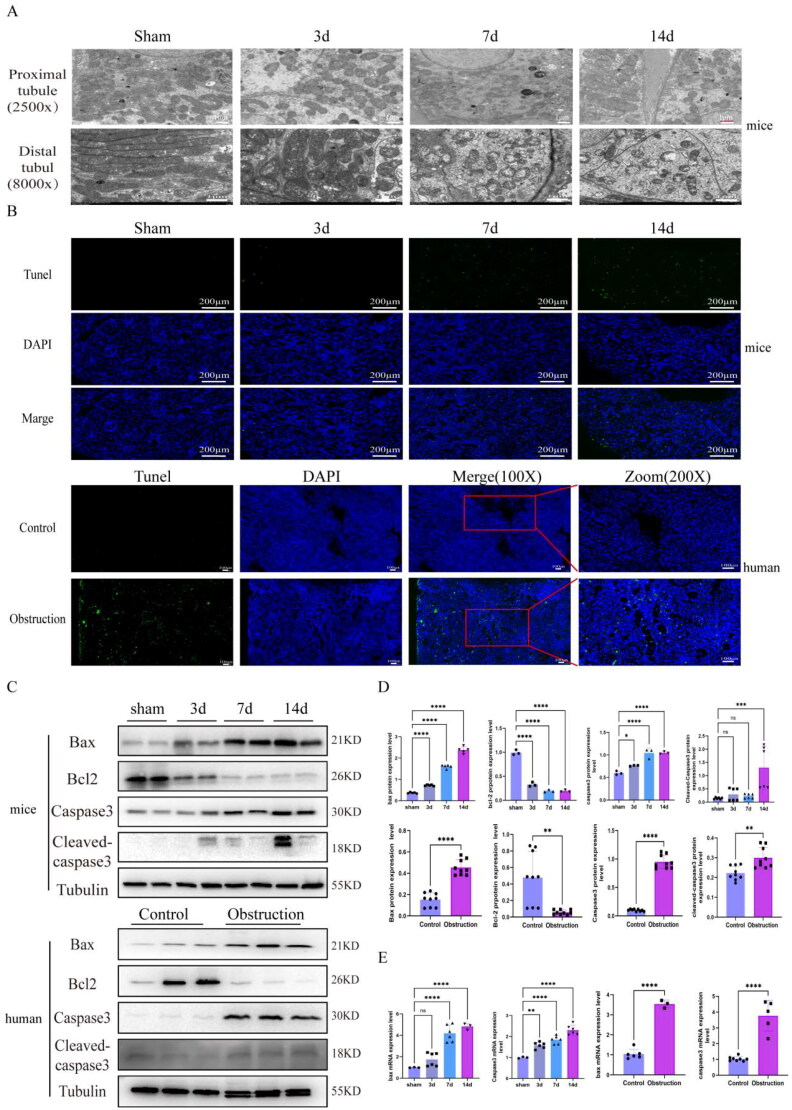
Mitochondrial dysfunction and apoptosis in mice UUO and human obstructed renal specimens. (A) The mitochondrial electron microscope results of renal proximal tubule (2500X, 8.0kv) and distal tubule (8000X, 8.0kv) in mice UUO model (*n* = 3 mice per group, 3 random sights per mice). (B) Tunel staining in mice UUO model and human obstructed renal specimens, the green points represented the apoptosis cell, the blue points represented the nucleus, Scale bar 200 μm, 100 μm (*n* = 5 mice per group, 4 random sights per mice). (C) Western blot of Bax, Bcl2, Caspase3, Cleaved-caspase3 in mice UUO and human obstructed renal specimens (*n* = 5 mice per group). (D) The relative proteins expression levels of Bax, Bcl2, Caspase3 and Cleaved-caspase3 in mice UUO and human obstructed renal specimens. (E) The quantitative results of the mRNA expression levels of Bax, Caspase3 in mice UUO and human obstructed renal specimens. Statistical analyses were analyzed by one-way ANOVA or 2-tailed student’s t test. *p ≤ 0.05, **p ≤ 0.01, ***p ≤ 0.001, ****p ≤ 0.0001.

We examined the mitochondrial structure with a transmission electron microscope and found that UUO model mice exhibited more mitochondrial ridge fracture and mitochondrial vacuolation than sham group in both the proximal tubules or distal tubules (Figure 4A). In addition, we examined the cell apoptosis level *via* TUNEL staining, and the results revealed that the degree of cell apoptosis increased in the kidneys of UUO model mice and in obstructed kidney specimens from humans (Figure 4B). Using TUNEL staining, we analyzed the proteins and mRNA expression of apoptosis-related markers in the kidneys of UUO model mice and in obstructed renal specimens from humans. We found that the proteins expression of Bax, Caspase3 and Cleaved-caspase3 increased in the kidneys of UUO model mice and in obstructed renal specimens from humans, the expression of Bcl2 decreased (Figure 4C). The quantitative results of apoptosis-related proteins were showed in Figure 4D. The mRNA level of Bax and Caspase3 also increased in the kidneys of UUO model mice and in obstructed kidney samples from humans (Figure 4E).

### Piezo1 facilitated the initiation of TCMK-1 cell injury and fibrosis

**Figure 5.**
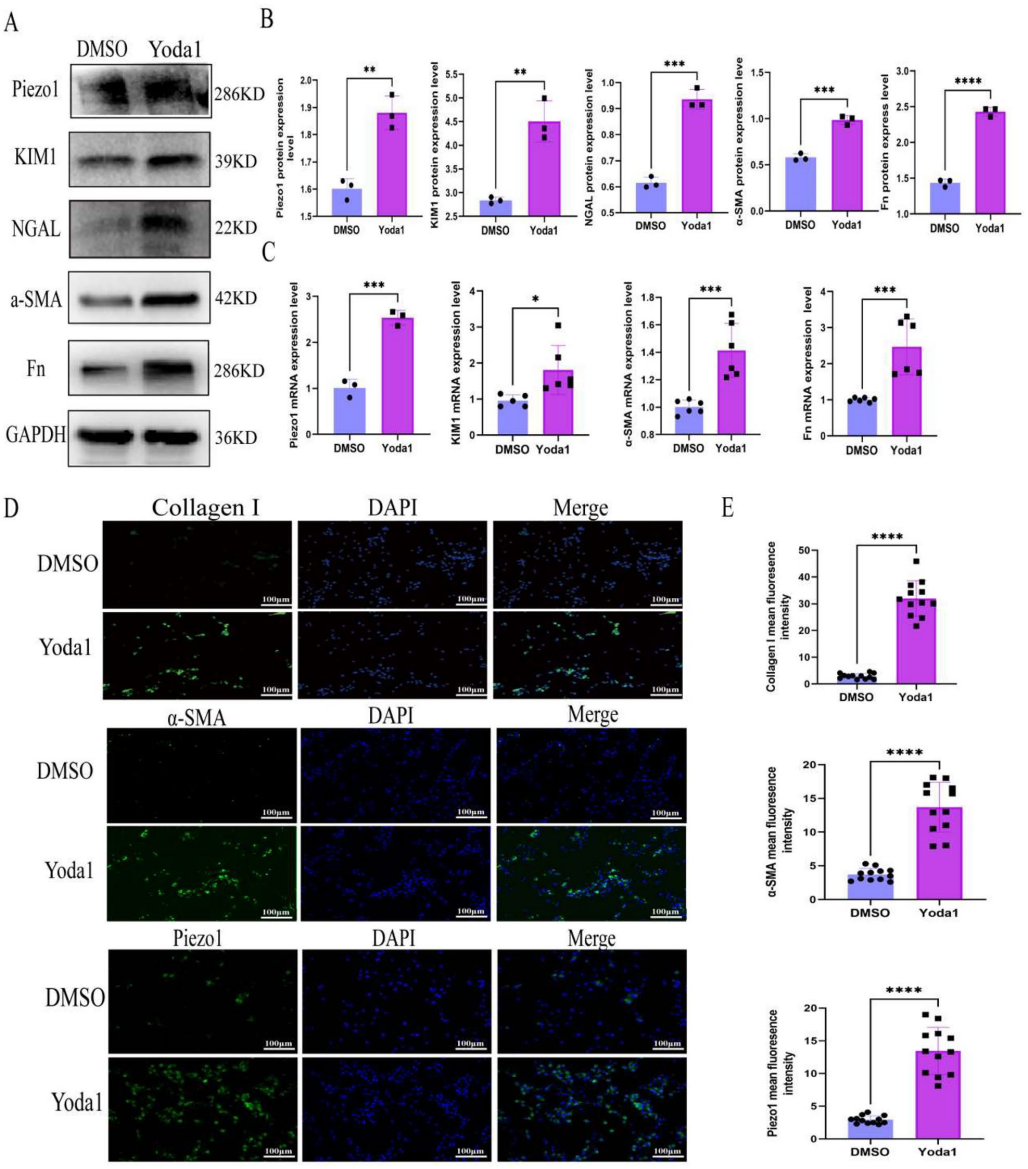
Yoda1 activatedPiezo1 channel to facilitate TCMK-1 cell injury and fibrosis. (A) The WB of Piezo1, Kim1, NGAL,α-SMA and Fn (*n* = 3 per group). (B) Bar plot represents the proteins quantification results of Piezo1, Kim1, NGAL, α-SMA and Fn. (C) Bar plot represents the mRNA quantification results of Piezo1, KIM1, α-SMA and Fn. (D) Immunostaining staining of Piezo1, α-SMA and Collagen I in TCMK-1 cell after Yoda1 treatment (*n* = 3 per group, 4 random sights per sample), Scale bar 100 μm. (E) The mean fluoresence intensity of Piezo1, α-SMA and collagen I. Statistical analyses were calculated using 2-tailed student’s t test for 2 groups. *p ≤ 0.05, **p ≤ 0.01, ***p ≤ 0.001, ****p ≤ 0.0001.

Based on the above results, we hypothesized that Piezo1 expression induced mitochondrial dysfunction and apoptosis to facilitate injury and fibrosis. To test the hypothesis, we conducted a Yoda1(specific Piezo1 agonist) intervention study. We performed the CCK8 assays and chose 4 µm as the suitable concentration for Yoda1 treatment (supplementary Figure 1A). After 4µm Yoda1 treatment, the protein expression levels of Piezo1, KIM1, NGAL, α-SMA and Fn increased (Figure 5A), and the quantitative analysis of relative proteins were shown in Figure 5B. Additionally, we found that the mRNA levels of Piezo1, NGAL, α-SMA and Fn increased (Figure 5C). Furthermore, we found that the fluorescence intensities and mean fluoresence intensities of Piezo1, α-SMA and Collagen I were higher after Yoda1 treatment than after DMSO treatment (Figure 5D and E).

### Piezo1 facilitated the initiation of TCMK-1 cell injury and fibrosis through Bax mediating cell apoptosis and mitochondrial dysfunction

**Figure 6.**
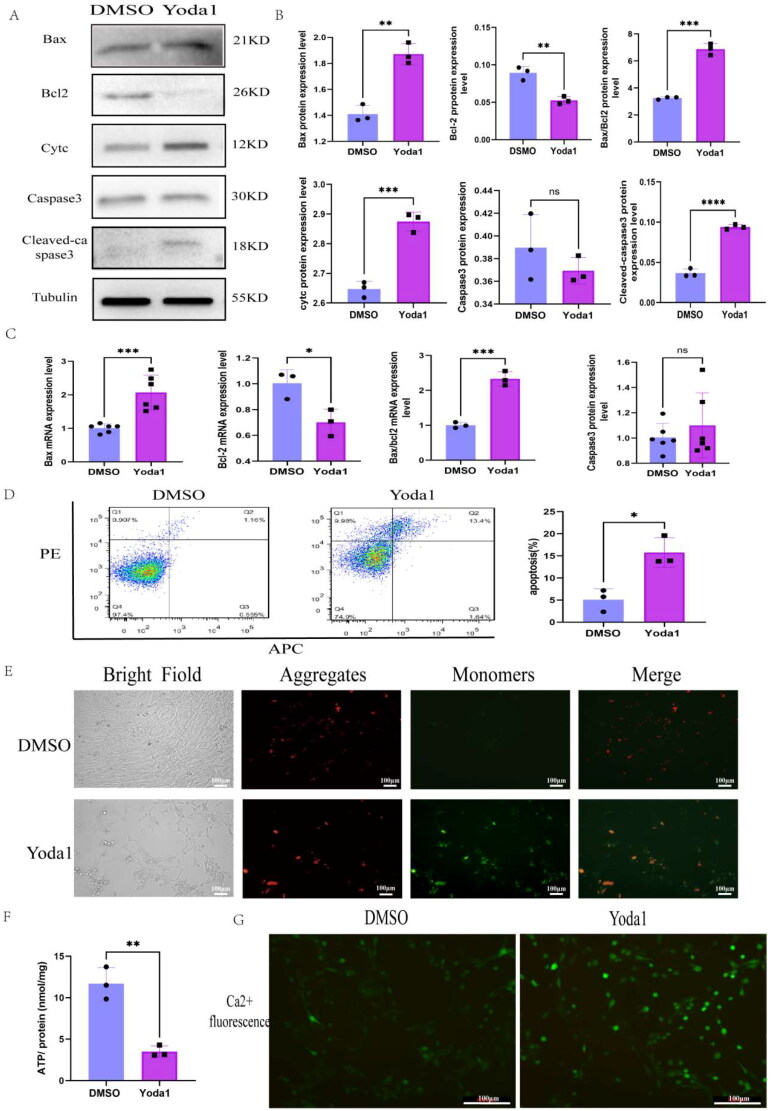
Yoda1 facilitated renal injury and fibrosis by Bax mediated apoptosis and mitochondrial dysfunction. (A) The WB of Bax, Bcl2, Cytc, Caspase3 and Cleaved-caspase3 (*n* = 3 per group). (B) Bar plot represents the proteins quantification results of Bax, Bcl2, Cytc, Caspase3 and Cleaved-caspase3. (C) The mRNA expression level of Bax, Bcl2, Bax/Bcl2 and Caspase3 after Yoda1 treatment. (D) Flow cytometry scatter plot of AnnexinV-PI/7ADD staining in TCMK-1 cells after Yoda1 intervention (*n* = 3 per group). (E) the immunostaining of mitochondrial membrane potential after Yoda1 treatment (*n* = 3 per group, 3 random sights per sample), Scale bar 100 μm. (F) Detection of intracellular ATP content in TCMK-1 cells after Yoda1 intervention. (G) the intracellular calcium concentration was detected with fluorescence microscopy after Yoda1 intervention, Scale bar 100 μm. Statistical analyses were calculated using 2-tailed student’s t test for 2 groups. *p ≤ 0.05, **p ≤ 0.01, ***p ≤ 0.001, ****p ≤ 0.0001

The above results show that Piezo1 facilitated the initiation of renal injury and fibrosis, but its mechanism is unclear. The role of mitochondrial in kidney physiology and pathology is crucial because of their involvement in the ATP production, the regulation of autophagy, stress responses and apoptosis [27]. However, whether the Piezo1 induces the initiation of renal injury and fibrosis by promoting tubular epithelium apoptosis is unknown. The WB results of apoptosis-related proteins Bax, Cytc, Caspase3 and Cleaved-caspase3 increased, and the expression of anti-apoptosis protein Bcl2 decreased after Yoda1 treatment (Figure 6A). The quantitative results for the proteins were similar to the WB results (Figure 6B). At the mRNA level, we observed increase of Bax, Bax/Bcl2, and Caspase3 but a decrease of Bcl-2 (Figure 6C). Similarly, we assessed the apoptosis level using flow cytometry and found that the apoptosis level increased after Yoda1 treatment (Figure 6D). Furthermore, we examined the mitochondrial membrane potential and intracellular ATP content, and found that the mitochondrial membrane potential and ATP content were decreased (Figure 6E and F). On the contrary intracellular calcium ([Ca2+]i) levels increased after Yoda1 treatment (Figure 6G).

**Figure 7.**
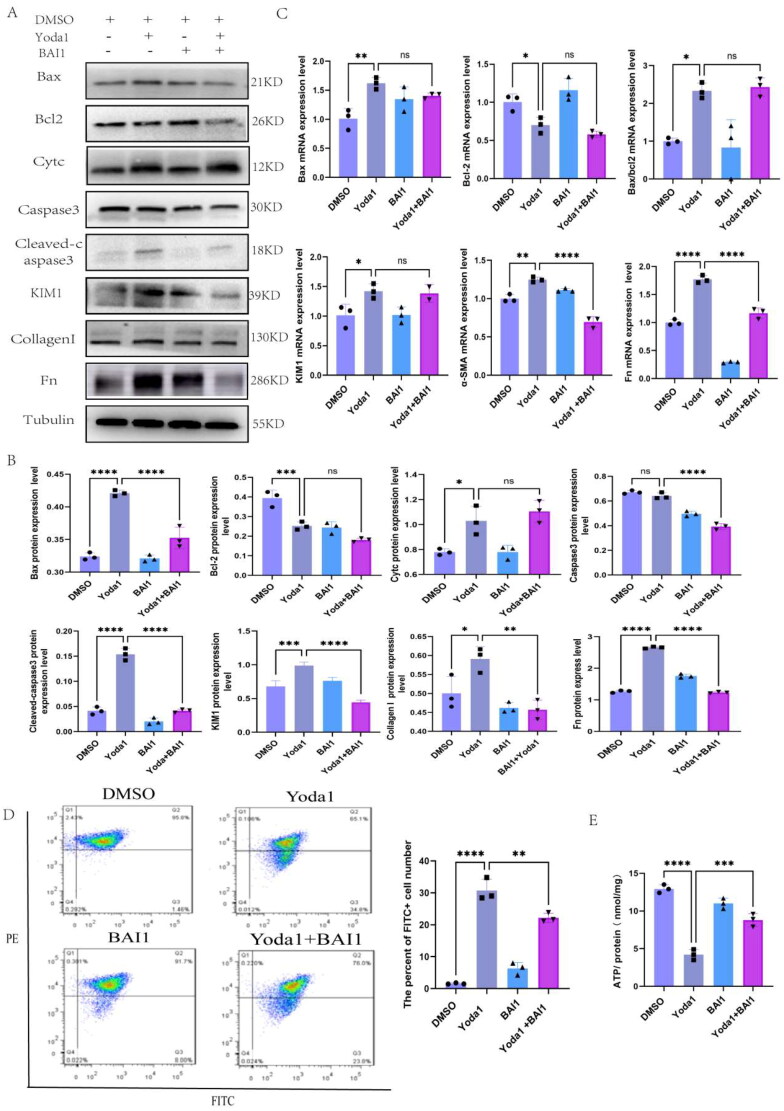
The rescue experiments that activated Piezo1 and inhibited Bax. (A) The WB expression levels of Bax, Bcl2, Cytc, Caspase3, Cleaved-caspase3, KIM1, Collagen I and Fn (*n* = 3 per group). (B) Bar plot represents the proteins quantification results of Bax, Bcl2, Cytc, Caspase3, Cleaved-caspase3, KIM1, Collagen1 and Fn. (C) The mRNA expression levels of Bax, Bcl2, Bax/Bcl2, α-SMA, Collagen I and Fn. (D) Flow cytometry scatter plot of JC1 staining after Yoda1 or/and BAI1 co-treatment, the FITC+ cells present the decreased of mitochondrial membrane potential (*n* = 3 per group). (E) Detection of intracellular ATP content in TCMK-1 cells after Yoda1 and/or BAI1 intervention. Statistical analysis was calculated using one-way ANOVA, *p ≤ 0.05, **p ≤ 0.01, ***p ≤ 0.001, ****p ≤ 0.0001.

We conducted a rescue experiment to verify whether Piezo1 mediated apoptosis and mitochondrial dysfunction through Bax. BAI1, a specific inhibitor of Bax, suppresses Bax-mediated apoptosis [28]. We performed the CCK8 assays and chose 4 µm as the suitable concentration of BAI1 treatment (supplementary Figure 1A). The TCMK-1 cells were treated with Yoda1 and/or BAI1, and the apoptosis, renal injury, renal fibrosis and mitochondrial function were assessed. The WB and qPCR results indicated that Bax, Cytc, Caspase3, Cleaved-caspase3, KIM1, Fn and Collagen I expression were higher in the Yoda1 treatment group than in the DMSO group but lower in the Yoda1 + BAI1 group than in Yoda1 group (Figure 7A and C). Figure 7B showed the quantitative analysis of the WB results. Additionally, we assessed the mitochondrial membrane potential using flow cytometry. There was a negative correlation between the proportion of FITC+ cells and membrane potential. The results indicated that the mitochondrial membrane potential decreased after Yoda1 treatment, but recovered after cotreatment with BAI1 (Figure 7D). In addition, the change in intracellular ATP content was similar with the change in mitochondrial membrane potential (Figure 7E).

### Targeting Piezo1 alleviated the development of renal injury and fibrosis

**Figure 8.**
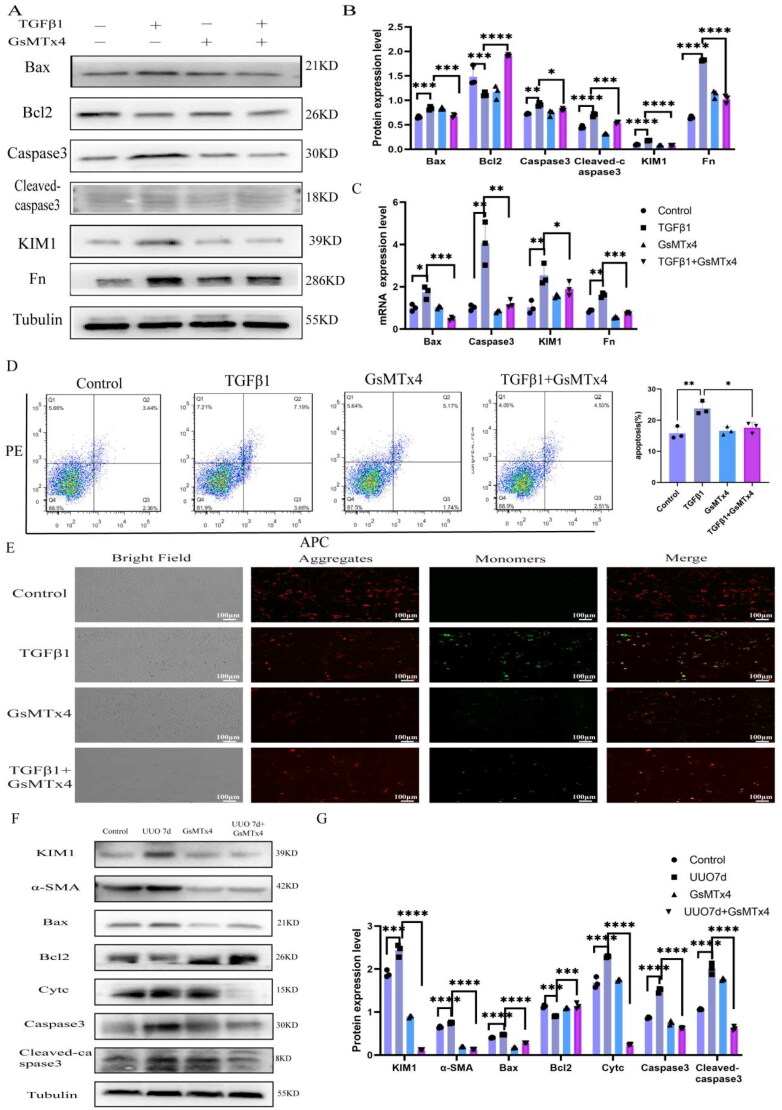
Inhibition Piezo1 could relief renal injury and fibrosis. (A) The WB expression levels of Bax, Bcl2, Caspase3, Cleaved-caspase3, KIM1 and Fn (*n* = 3 per group). (B) The WB quantitative results of Bax, Bcl2, Caspase3, Cleaved-caspase3, Kim1 and Fn. (C) The mRNA level of Bax, Caspase3, KIM1 and Fn. (D) Flow cytometry scatter plot of AnnexinV-PI/7ADD staining in TCMK-1 cells after TGFβ1 and/or GsMTx4 intervention (*n* = 3 per group). (E) The immunostaining of mitochondrial membrane potential after TGFβ1 and/or GsMTx4 in intervention (*n* = 3 per group, 3 random sights per sample), JC1 aggregates (red), JC1 monomers (green), Scale bar 100 μm. (F) The WB expression levels of Kim1, α-SMA, Bax, Bcl2, Cytc, Caspase3 and Cleaved-caspase3 in UUO mice models intervened by GsMTx4. (G) The WB quantitative results of KIM1,α-SMA, Bax, Bcl2, Cytc, Caspase3 and Cleaved-caspase3 in UUO mice models intervened by GsMTx4. Statistical analyses were calculated using one-way ANOVA. *p ≤ 0.05, **p ≤ 0.01, ***p ≤ 0.001, ****p ≤ 0.0001.

We demonstrated that Piezo1 activation promoted the initiation of TCMK-1 injury and fibrosis through the Bax/caspase3 pathway, but whether Piezo1 can be an intervention target in the development of CKD fibrosis is unclear. GsMTx4, an inhibitor of Piezo1, can be used to study the function of Piezo1 *in vivo* and *in vitro* [29]. We performed the CCK8 assays and chose 4 µm as the suitable concentration of GsMTx4 treatment (supplementary Fig.1B). We stimulated cells with 10 ng/ml TGFβ1 and divided into four groups (Control, TGFβ1, GsMTx4, and TGFβ1 + GsMTx4). The results showed GsMTx4 treatment reversed the TGFβ1-induced upregulation of Bax, Caspase3, Cleaved-caspase3, KIM1, and Fn expression (Figure 8A). The quantitative results for related-proteins were presented in Figure 8B. The gene expression levels of Bax, Caspase3, Kim1 and Fn were consistent with the protein levels (Figure 8C). Similarly, we demonstrated that the TGFβ1-induced increase in the apoptosis level and intracellular calcium ([Ca2+]i) level were recovered after GsMTx4 treatment (Figure 8D, supplementary Figure 2B). Additionally, we examined the change in mitochondrial membrane potential and intracellular ATP content. The results showed that GsMTx4 treatment reversed the TGFβ1-induced decrease in mitochondrial membrane potential and ATP content (Figure 8E, supplementary Figure 2A). In addition, a suppression experiment using GsMTx4 was conducted in mice *in vivo*. The results showed that the increases in renal injury, fibrosis and apoptosis levels induced by UUO were partially prevented by GsMTx4 treatment (Figure 8F–G).

## Discussion

CKD has become a major global public health issue and contribute to increasing morbidity and mortality [30]. UUO, one of the causes of CKD, involves hemodynamic alterations, oxidative stress, apoptosis, and inflammation which ultimately lead to fibrosis development [31]. Renal fibrosis is the most prominent hallmark of CKD, and the main approach of delaying CKD is to suppress renal fibrosis [32]. Although many studies have explored the mechanisms of fibrosis, it is unclear how the cells and the mechanical sensitive protein Piezo1 interact during fibrosis progression.

In the study, we demonstrated that Piezo1 played a key role in the initiation and progression of renal injury and fibrosis. We found that Piezo1 activation could induced the TCMK-1 cells injury and fibrosis through apoptosis and mitochondrial dysfunction. In addition, GsMTx4 protected TCMK-1 cells from TGFβ1-induced injury and fibrosis by decreasing apoptosis and mitochondrial dysfunction.

Recent studies have suggested that the biomechanical properties of cells, especially the structural and mechanical properties of cells as well as abnormal mechanotransduction, are related to disease [33]. Parenchymal cells and nonparenchymal cells are subjected to different mechanical stimuli during chronic injury, which is considered both a consequential and causative factor of fibrotic diseases [34]. Mechanical stimulation also plays an important role in the phenotypic transition of fibroblasts [35], in stem cell fate, behavior and developmental processes [36] and in enhancement of bone regeneration [37]. Cellular mechanical stimuli include curvature, stiffness, strain, fluid shear stress and hydrostatic pressure [37]. In addition, epithelium cells can sense changes caused by mechanical stimulation and transduce external mechanical signals to intracellular chemical and electronic signals *via* mechanoreceptors ion channels, integrins, G protein–coupled receptors, glycocalyx, to regulate the cell proliferation, differentiation, and apoptosis [23]. Piezo1 is a mechanoreceptor that has been shown to be widely expressed in the urinary system [15]. In the kidney, Piezo1 is expressed in glomerular endothelial cells, parietal cells of Bowman’s capsule, distal convoluted tubule endothelial cells, principal cells of collecting ducts, urothelial cells of the renal pelvis and proximal tubules [15,38]. In obstructive nephropathy, postobstruction hydrostatic pressure is elevated, which leads to the abnormal mechanical, microenvironmental, and stretch/compression stimulation of tubular epithelial cells. This aberrant stimulation can be responded by mechanosensors Piezo1, which is located on the basolateral plasma membrane [23], and converts mechanical stimulation into cell and tissue injury signals. Therefore, tubular epithelial cells are the primary effector cells of obstructive nephropathy. In our study, we found that Piezo1 was expressed in the proximal tubules of UUO model mice and obstructed kidneys samples from humans, and that the abundance of Piezo1 mRNA and protein were positively associated with renal injury and renal fibrosis, this is consistent with previous reports [23,24]. Thus, we hypothesized that Piezo1 participates in renal injury and the progression of fibrosis.

To explore the role of Piezo1 in the initiation of renal injury and fibrosis, we performed *in vitro* experiments. Yoda1, a widely used synthetic small molecule Piezo1 activator, can effectively lower the mechanical threshold of Piezo1 channel activation by stabilizing the open conformation of the channel [39,40]. This makes Yoda1 partially activate Piezo1 channels in the absence of mechanical stimulation [40]. Based on the function of Yoda1, we treated the TCMK-1 cells with Yoda1 alone and found that the mRNA and protein expression of Piezo1, fibrosis markers and injury markers increased. In contrast, Zhao X [23] found Yoda1 treatment caused renal fibrosis but did not affect the Piezo1 abundance. One reason for this discrepancy may be that the mechanism by which Yoda1 activates Piezo1 is not fully understood. Although opinions differ on whether Yoda1 can regulate the expression of Piezo1, there is a consensus that Yoda1 treatment induces fibrosis. Therefore, the Piezo1 activation could be a trigger for fibrosis.

Whether mitochondria are involved in the initiation of renal injury and fibrosis is unknown. In the experiment of Yoda1 intervention experiments, TCMK-1 cells apoptosis increased, and the mitochondrial membrane potential and ATP content decreased. Mitochondria are productive energy organs, and researchers have reported that mitochondrial damage plays a key role in tubular cell injury [41]. When stimulated, mitochondria swell and fragment, which lead to reduced ATP production, increased ROS production, disturbance of cytochrome C release and distribution, cytokines production and cellular death (necrosis and apoptosis), these pathological changes lead to initial damage of tubular cell injury [41,42]. Accordingly, mitochondria may serve as effective targets for injury treatment. Bax is a proapoptotic protein that can be recruited into mitochondria member and induce mitochondrial membrane potential depolarization and cell apoptosis [43,44]. Furthermore, Piezo1 downregulation induces esophageal squamous cell carcinoma apoptosis *via* thePiezo1-p53-Bax-Caspase 3 axis [45]. In contrast, the expression of Piezo1, Bax and Caspase3 increased, the level of cells apoptosis increased, and mitochondrial damaged occurred after Yoda1 treatment in this study. Therefore, we determined that increased Piezo1expression led to cell injury and further fibrosis through Bax. To confirm those results, we introduced the Bax inhibitor BAI1, which inhibits Bax-dependent cell death by preventing Bax mitochondrial translocation and oligomerization [28]. We treated TCMK-1 cells with Yoda1 and/or BAI1, and found that the degree of cell apoptosis, mitochondrial damage and fibrosis was lower in the cotreatment group than in the Yoda1 alone group. Therefore, we hypothesized that Piezo1 induces renal injury and fibrosis through Bax mediated apoptosis and mitochondrial dysfunction.

Although we have shown that Piezo1 can induce the initiation of kidney injury and fibrosis through Bax-mediated apoptosis and mitochondrial dysfunction, it is unclear whether the inhibition of Piezo1 can alleviate the development of kidney injury and fibrosis. GsMTx4, a gating modifier peptide from spider venom [29], can reduce the membrane tension near the Piezo1 channel by inserting into the lipid bilayer, and inhibit the transition of the Piezo1 channel from closed to open, thus producing a nonspecific inhibitory effect [46,47]. Studies have demonstrated that Piezo1 can induce the apoptosis of pancreatic cancer cells [48], pneumocytes [17] and nucleus pulposus cells [49], and the inhibition of Piezo1 can treat apoptosis-related diseases. In our study, we treated cells with GsMTx4 *in vitro* or *in vivo* and found that the inhibition of Piezo1 decreased the degree of cells apoptosis, restored mitochondrial function and protected against renal injury and fibrosis. This finding is consistent with the results reported by Wang et al. [50]. Thus, targeting Piezo1 may be an effective treatment for alleviating renal injury and fibrosis.

In conclusion, Piezo1 plays an essential role in renal injury and fibrosis. Piezo1 promotes the initiation of renal injury and fibrosis *via* Bax-mediated apoptosis and mitochondrial dysfunction, and the inhibition of Piezo1 suppresses the progress of CKD fibrosis. These results emphasize the importance of Piezo1 in the renal fibrosis.

However, there are still some limitations in this study. First, the expression of piezo1 and the degree of cell apoptosis under different hardness were not studied. In addition, studies in which mechanical forces induce Piezo1, apoptosis, and fibrosis are lacking. Thus, it needs to further research.

## Supplementary Material

Supplement.docx

Graphical Abstract.docx

## Data Availability

Data will be made available on request.
