# Supplementary material for: Piezo1 facilitates the initiation and progression of renal fibrosis by mediating cell apoptosis and mitochondrial dysfunction
Source: Ren Fail. 2024 Nov 4;46(2):2415519. doi: 10.1080/0886022X.2024.2415519 (PMC11536639; doi:10.1080/0886022X.2024.2415519)
Supplement: Supplement.docx [file IRNF_A_2415519_SM4724.docx]

Supplementary materials

Table1 Basic characteristics of patients

| Group | Sex | Age  (year） | years since diagnosis with renal carcinoma(month) | past medical history | Medications | Renal  glomerular filtration  rate on  injury side  (ml/min) | Clinical Diagnosis |
| --- | --- | --- | --- | --- | --- | --- | --- |
| Control 1 | M | 27 | 11 | no | no | 60 | Kidney cancer |
| Control 2 | M | 36 | 8 | no | no | 84 | Kidney cancer |
| Control 3 | F | 42 | 8 | appendectomy | no | 76 | Kidney cancer |
| Control 4 | M | 50 | 9 | pneumonia | no | 89 | Kidney cancer |
| Control 5 | M | 48 | 9 | no | no | 90 | Kidney cancer |
| Obstruction 1 | M | 30 | 7 | Left ureteral stone | no | 10 | Left  hydronephrosis |
| Obstruction 2 | M | 36 | 6 | Left ureteral stone | no | 14 | Left  hydronephrosis |
| Obstruction 3 | M | 24 | 7 | no | no | 15 | Right hydronephrosis |
| Obstruction 4 | M | 58 | 5 | no | no | 20 | Left  hydronephrosis |
| Obstruction 5 | F | 48 | 9 | no | no | 16 | Right hydronephrosis |

Table 2 Mouse and human mRNA primer sequences

| name | sequences |
| --- | --- |
| 1. piezo1 -S | AGCATTGACTTTCACCGCAGG |
| H-piezo1- A | TGCTTGGCACGGATACGCT |
| H-BCL2(RZ)-S | GGAGGATTGTGGCCTTCTTTG |
| H-BCL2(RZ)-A | GCATCCCAGCCTCCGTTATC |
| H-BAX-S | TTTTGCTTCAGGGTTTCATCCA |
| H-BAX-A | TGCCACTCGGAAAAAGACCTC |
| H-lcn2(1)-S | GACAACCAATTCCAGGGGAAG |
| H-lcn2(1)-A | CAGGACGGAGGTGACATTGTAG |
| H-kim-S | TTCACCATCTTCACCTCAGCCA |
| H-kim-A | TCACGGTGTCATTCCCATCTGT |
| H-COL1A1-S | CCAAGACGAAGACATCCCACCA |
| H-COL1A1-A | CCGTTGTCGCAGACGCAGAT |
| H-FN1（1）-S | GGAGAGTGGAAGTGTGAGAGGC |
| H-FN1（1）-A | TCCATTTGAGTTGCCACCGT |
| H-α-SMA-S | CAATGTCCTATCAGGGGGCAC |
| H-α-SMA-A | CGGCTTCATCGTATTCCTGTT |
| H-CASP3-S | AGAACTGGACTGTGGCATTGAG |
| H-CASP3-A | CACAAAGCGACTGGATGAACC |
| 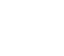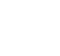H-ACTIN-S | 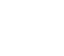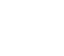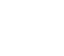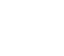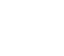CACCCAGCACAATGAAGATCAAGAT |
| H-ACTIN-A | CCAGTTTTTAAATCCTGAGTCAAGC |
| M-Piezo1-S | AGCCATCCATTGTGCCATT |
| M-Piezo1-A | TCCAAGGTGTGCTTCTCATTAGTAT |
| M-BCL2-S | TGACTTCTCTCGTCGCTACCGT |
| M-BCL2-A | CCTGAAGAGTTCCTCCACCACC |
| M-KIM(1)-S | CCTATCAGAAGAGCAGTCGGTACA |
| M-KIM(1)-A | TGGATCTTGTTGAAATAGTCGTGG |
| M-LCN2-S | ACAGAAGGCAGCTTTACGATGT |
| M-LCN2-A | ACTGGTTGTAGTCCGTGGTGG |
| M-CASP3- S | TGGAATGTCATCTCGCTCTGGT |
| M-CASP3-A | GAAGAGTTTCGGCTTTCCAGTC |
| M-Bax-S | GCCTTTTTGCTACAGGGTTTCAT |
| M-Bax-A | TATTGCTGTCCAGTTCATCTCCA |
| M-FN1(RZ)- S | AAGGCTGGATGATGGTGGACT |
| M-FN1(RZ)- A | TCGGTTGTCCTTCTTGCTCC |
| M-col1a1 -S | GAGAGGTGAACAAGGTCCCG |
| M-col1a1 -A | AAACCTCTCTCGCCTCTTGC |
| M-α-SMA(2)-S | GTACCACCATGTACCCAGGC |
| M-α-SMA(2)-A | GAAGGTAGACAGCGAAGCCA |
| M-β-actin-S | GTGACGTTGACATCCGTAAAGA |
| M-β-actin-A | GTAACAGTCCGCCTAGAAGCAC |

A B

**Fig 1. The results of TCMK-1 cells CCK8 with Yoda1, BAI1 and GsMTx4.** **A** The CCK8 experiment of different concentrations Yoda1 and BAI1 intervention. **B** The CCK8 experiment of different concentrations GsMTx4 intervention.


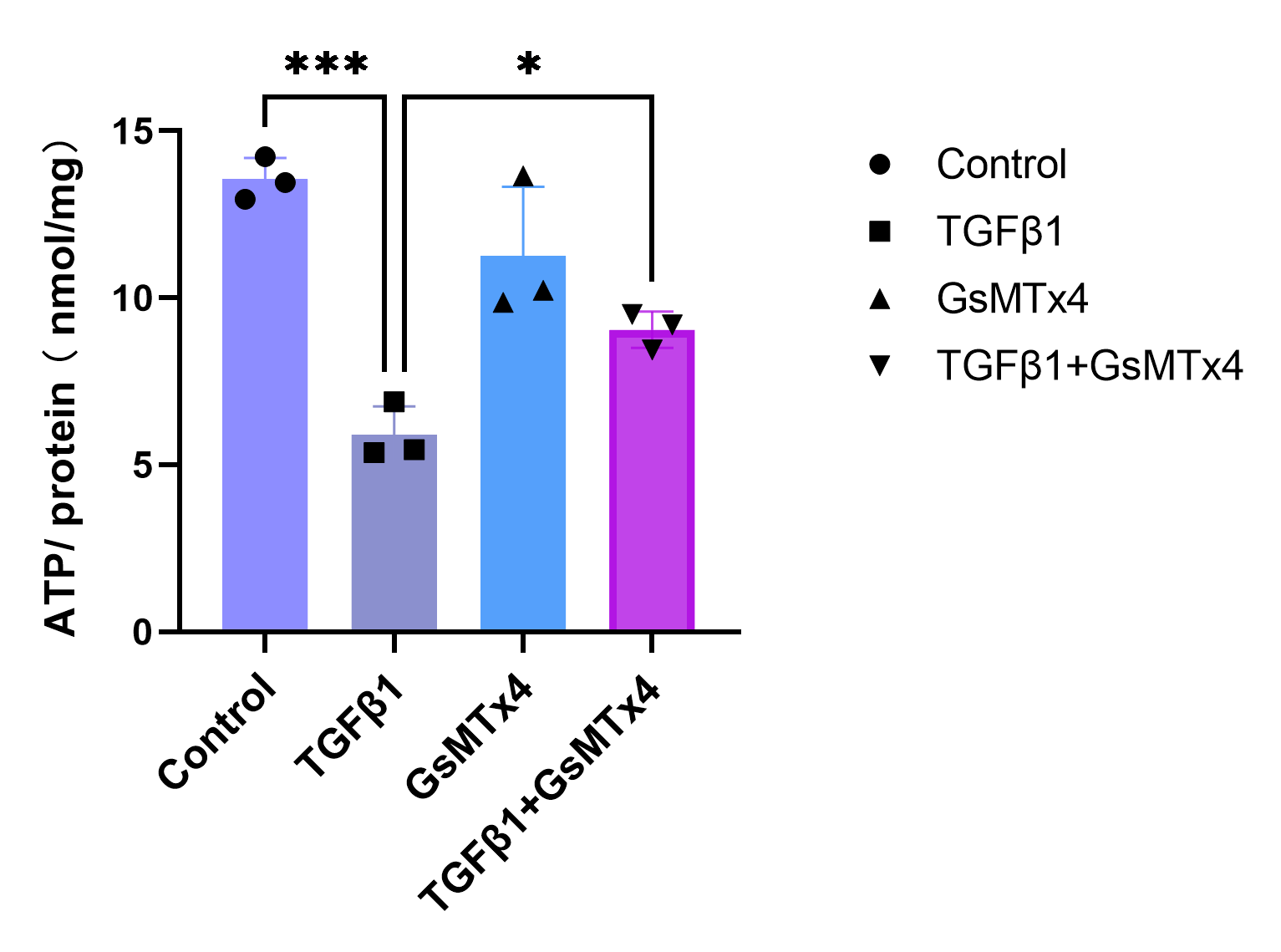
 A

B


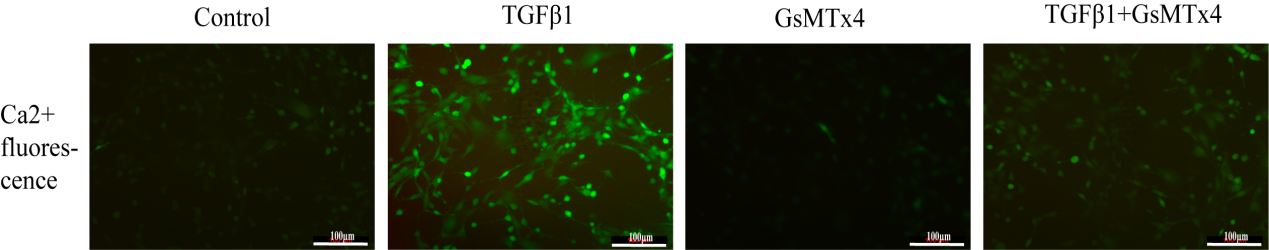


**Fig2. A** Detection of intracellular ATP content in TCMK-1 cells after TGFβ1 and/or GsMTx4 intervention. **B**

The intracellular calcium concentration were detected with fluorescence microscopy in TCMK-1 cells after TGFβ1 and/or GsMTx4 intervention, Scale bar 100 μm.
