# Supplementary material for: Piezo1 facilitates the initiation and progression of renal fibrosis by mediating cell apoptosis and mitochondrial dysfunction
Source: Ren Fail. 2024 Nov 4;46(2):2415519. doi: 10.1080/0886022X.2024.2415519 (PMC11536639; doi:10.1080/0886022X.2024.2415519)
Supplement: Graphical Abstract.docx [file IRNF_A_2415519_SM4723.docx]

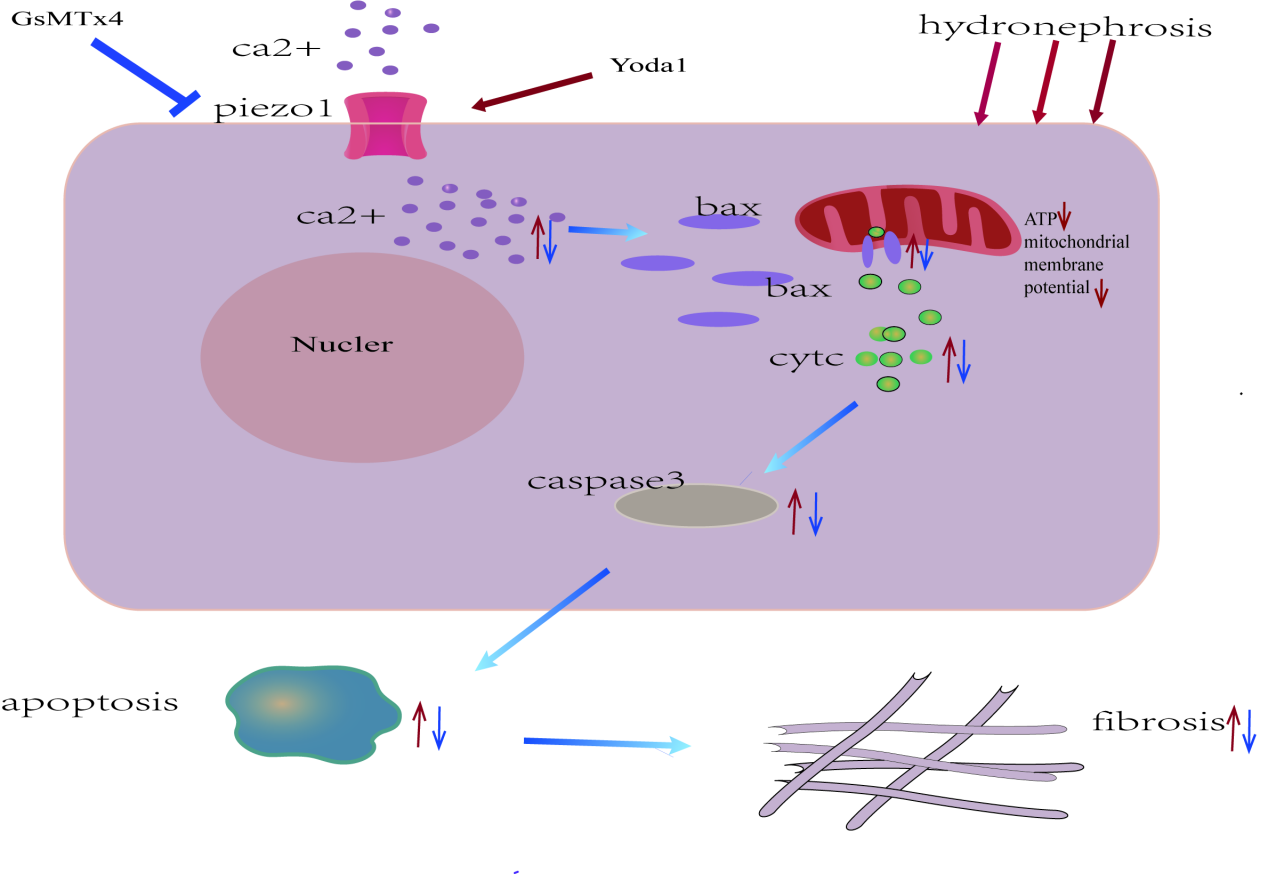


**Figure legend:** **The signal path diagram of Piezo1 inducing real injury and fibrosis.** The expression of mechanically sensitive channel Piezo1 increased in renal proximal tubular epithelial cells during hydronephrosis. The increased Piezo1 permeated extracellular Ca2+ into intracellular, then facilitated the translocation of Bax to the mitochondrial membrane, which decreased mitochondrial membrane potential and leaded to Cytc leakage, and facilitated the apoptosis, then induced the fibrosis. Inhibition Piezo1 using GsMTx4 can improve the apoptosis and fibrosis.
